# The impact of location and patency of the arteriovenous fistula on quality of life of kidney transplant recipients

**DOI:** 10.1080/0886022X.2020.1865171

**Published:** 2021-01-05

**Authors:** Krzysztof Letachowicz, Klaudia Bardowska, Tomasz Królicki, Dorota Kamińska, Mirosław Banasik, Karolina Zajdel, Oktawia Mazanowska, Katarzyna Madziarska, Dariusz Janczak, Magdalena Krajewska

**Affiliations:** a Department of Nephrology and Transplantation Medicine, Wroclaw Medical University, Wroclaw, Poland; b Faculty of Medicine, Wroclaw Medical University, Wroclaw, Poland; c Department of Vascular, General and Transplantation Surgery, Wroclaw Medical University, Wroclaw, Poland

**Keywords:** Hemodialysis, arteriovenous fistula, kidney transplantation, quality of life

## Abstract

**Background:**

Arteriovenous fistulae (AVFs) may remain patent after kidney transplantation (KTx), contributing to maladaptive cardiac remodeling. The flow in AVFs is associated with the diameter of its vessels and thus with the AVF location. The main objective of this study is to assess the influence of AVF location and its patency on the self-reported quality of life (QOL) of kidney transplant recipients (KTRs) with past history of hemodialysis.

**Methods:**

To gain clinical data, during a scheduled visit, 353 KTRs were asked to fill out an anonymous questionnaire. From this group, 284 respondents were found eligible for analysis. The outcome was defined as prevalence of symptoms and health status, measured with the Left Ventricular Dysfunction-36 (LVD-36) Questionnaire in symptomatic patients.

**Results:**

The hemodialysis patients (*n* = 243) were divided into two groups according to AVF location, i.e., DAVF – distally located AVF – (*n* = 174) and PAVF – proximally located AVF – (*n* = 69). The proportion of patients with heart failure (HF) was higher in PAVF group (24% vs. 12%, *p* = 0.0482). In the multivariable regression, PAVF, serum creatinine levels, and the presence of HF or coronary artery disease (CAD) remained independent predictors of lower functional capacity. Among patients with heart disease, the presence of active AVF was independently associated with worse functional outcome (higher LVD-36 scores).

**Conclusions:**

The influence of persistent PAVF in KTRs seems to be unfavorable, especially when coexisting with CAD or HF. **Abbreviations:** AVF arteriovenous fistula; BMI body mass index; CAD coronary artery disease; D-AVF distally-located arteriovenous fistula; EC exercise capacity; HD hemodialysis; HF heart failure; KTx kidney transplantation; KTR kidney transplant recipient; LVD-36 Left Ventricle Disfunction – 36; LVEF left ventricle ejection fraction; LVH left ventricle hypertrophy; NYHA New York Heart Association; P-AVF proximally located arteriovenous fistula; PD peritoneal dialysis; PRO patient-reported outcomes; QOL quality of life.

## Introduction

Kidney transplantation (KTx) is a method of choice in treatment of end-stage renal disease [1,2]. It reduces cardiovascular mortality and it is associated with better cardiopulmonary capacity and quality of life (QOL) compared to dialysis patients [3–5]. Exercise capacity (EC) is one of the many factors which can significantly limit QOL after KTx, especially in young patients who want to remain active. Vascular access and vasculature preservation are crucial elements of care provided to the patients with chronic kidney disease after transplantation [6]. The influence of persistent arteriovenous fistula (AVF) on the condition of kidney transplant recipients (KTRs) with past history of hemodialysis is a controversial topic of debate. Several studies have already shown that the persistent AVF in this clinical setting leads to ongoing maladaptive cardiac remodeling, which seems to be at least partially reversible after AVF ligation [7–9]. Multiple studies have also revealed a positive correlation among AVF flow, cardiac output, and diastolic dysfunction severity, which is a burden for patients with an already underlying cardiovascular disease [10]. Furthermore, proximally located AVF have been shown to present higher flow than the distal ones, as well as they are related to more severe cardiac remodeling [11]. On the other hand, some authors suggest that the ligation of active AVF may be associated with accelerated decline of kidney function [12]. Additionally, the opinion regarding the potential AVF closure after kidney transplantation is inconsistent among professionals [13]. According to the guidelines of the European Society for Endovascular Surgery, a routine closure of a functioning vascular access after successful kidney transplantation is not routinely recommended [14]. We also lack precise data about the impact of AVF ligation or thrombosis on the cardiovascular risk, long-term prognosis, and quality of life in this patient group.

The main objective of this study was to assess the influence of persistent AVF and its location on the presence of symptoms of heart failure (HF) and functional well-being among symptomatic KTRs.

## Materials and methods

### Study design and data collection

**Figure 1.**
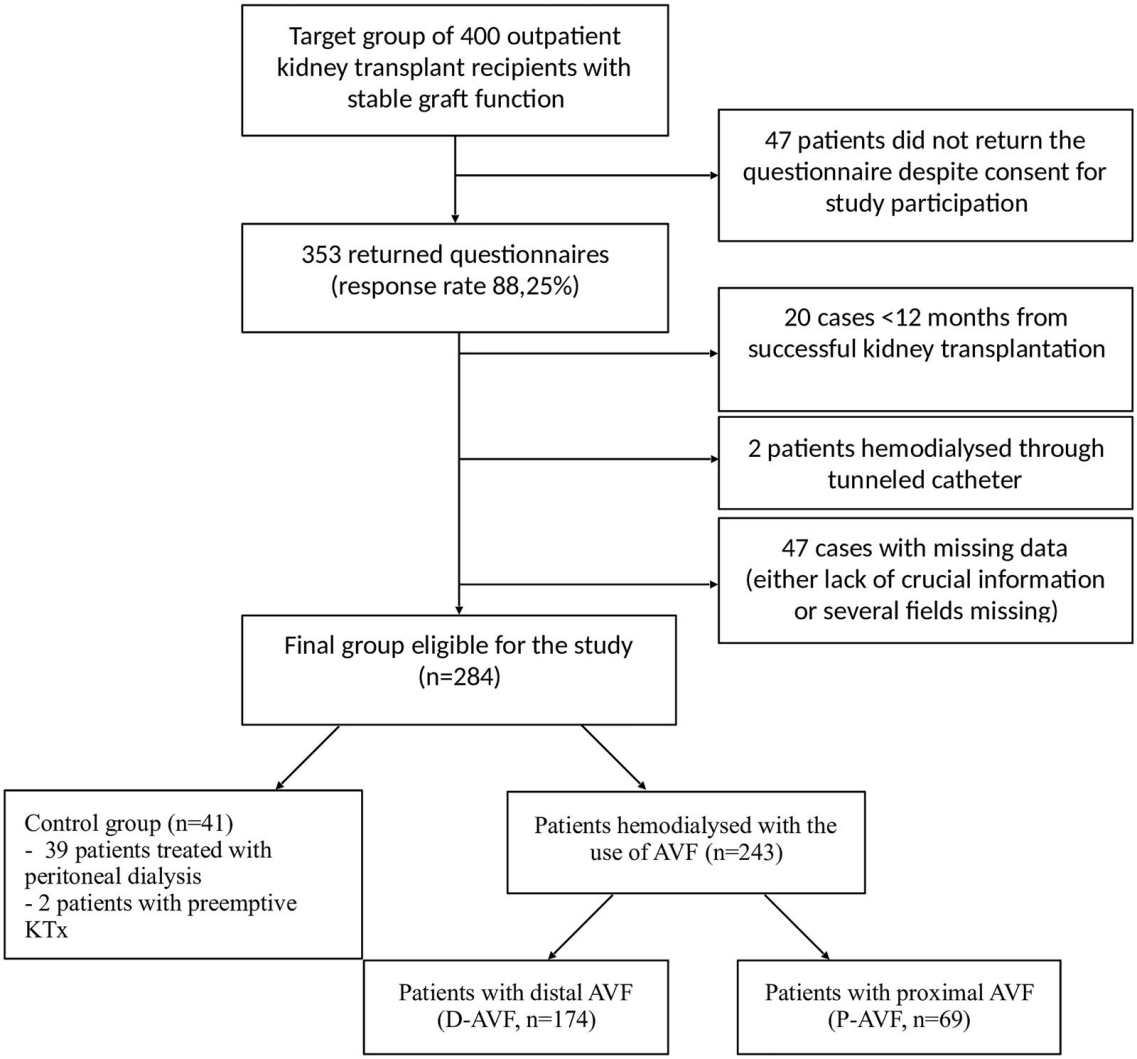
Flowchart presenting the process of study group selection and its structure.

The study was designed as a questionnaire-based, single-center, cross-sectional study. We have aimed to recruit 400 adults, outpatient kidney transplant recipients, more than 12 months after the transplantation. The questionnaire was anonymous and its return rate equaled 88.25% − 353 patients had filled it out and thus were included in the study. To avoid misreporting of data, all patients were instructed to fill out the questionnaire relying on their past medical records. In 47 cases important data were lacking. Patients were grouped according to dialysis modality, patency, and location of AVF. Only data of patients more than 12 months after transplantation were analyzed (20 cases were thus excluded). Two patients receiving dialysis through tunneled catheters were not included either. The process of study group selection and its structure is presented in the flowchart in Figure 1.

### Inclusion criteria

- Outpatient kidney transplant recipient (aged 18 or more);
- Willingness to participate in the survey.

### Exclusion criteria

- Cognitive impairment;
- Infection;
- HF in NYHA IV class;
- Rapid deterioration of kidney function.

### Questionnaire

The questionnaire included queries regarding patients’ medical history, demographic data, comorbidities, and prevalence of symptoms. Patent AVF was defined as presence of palpable thrill in the area of vascular anastomosis. Patients were asked about five types of complaints: dyspnea, peripheral edema, fatigue, worse exercise tolerance, and palpitations. On the basis of self-reported symptoms, the patients were classified either symptomatic (at least one complaint present) or asymptomatic. All symptomatic patients have also filled out the Left Ventricular Dysfunction-36 (LVD-36) Questionnaire to quantitate the disease burden and functional impairment. LVD-36 Questionnaire, designed to assess the health status and the severity of cardiovascular symptoms in patients with left ventricular impairment, shows a strong negative correlation with maximal oxygen uptake (VO_2_ max) and a positive correlation with New York Heart Association (NYHA) Classification, irrespectively from the etiology of dysfunction, left ventricular ejection fraction or other echocardiographic parameters [15]. Furthermore, it was also found to be highly repeatable and consistent with patients’ perception of exercise capacity. Therefore, LVD-36 score was used in the study for the measurement of QOL. Permission to use LVD-36 Questionnaire was obtained from St George’s University of London.

### Outcome

The measures were the number of symptoms of heart disease and LVD-36 score (given as percent of 36 points) in symptomatic patients. We hypothesized LVD-36 score as a mean of QOL quantification (higher scores indicate worse condition).

### Statistical analysis

The distribution of continuous variables was assessed with the use of Saphiro–Wilk test. Continuous data were presented, as appropriate, as median and interquartile ranges (IQR) or mean ± standard deviation (SD). The significance of differences between these data was tested using independent Student’s *t*-test or Mann–Whitney *U* test. The *p* values obtained in Table 3 for cross-comparison between groups were multiplicity-corrected (multiplied 10 times). Descriptive data are presented as frequencies and percentages. To compare them, chi-square tests or Fisher’s exact tests were performed as appropriate. A multivariable linear regression model, with stepwise backward elimination of variables, was performed to assess the impact of multiple factors on QOL. A two-tailed *p* value of <0.05 was statistically significant. All analyses were performed using Statistica 13.2 (StatSoft, Tulsa, OK, USA).

**Table 3.** Multivariable logistic regression model for predictors of symptoms’ occurrence in the whole study group.

	Univariable (*p* value)	OR	Multivariable (*p* value)
Age [years]	0.0005	1.02	0.0471
Obesity	0.0456	1.51	0.0249
Diabetes	0.0268	1.06	0.7181
Heart failure	0.0009	3.95	0.0081
Coronary artery disease	0.0002	2.68	0.0106

### Statement of ethics

The research was conducted ethically in accordance with the World Medical Association Declaration of Helsinki. The study was approved by the local ethics committee of the Wroclaw Medical University (approval number: KB-775/2018). All patients approved the Informed Consent Form.

## Results

Among 284 included patients, 243 had hemodialysis history (HD-group), 39 were treated with peritoneal dialysis (PD) and 2 received preemptive kidney transplantation. If the patients received both types of dialysis, they were assigned to HD-group, provided that HD preceded transplantation directly and the patients had had AVF created. The respondents receiving dialysis through tunneled catheters were not included due to small sample size and different characteristics. The patients who were treated with PD and those who received preemptive KTx were assigned to a control group (as these respondents are known to present better condition compared to their HD counterparts). Only five patients in the whole study group have admitted the presence of symptoms associated with patent AVF (mostly pain and extremity ischemia). These complaints were not severe enough for the patients to decide for ligation though. In patients without patent AVF, the access was ligated in three respondents and in the remaining cases, the AVF was lost in the course of thrombosis.

In Table 1, HD-group was compared to controls. The number of symptomatic patients did not differ between groups, but HD-group presented a significantly higher LVD-36 score among symptomatic respondents. This cohort was then divided into two subgroups according to AVF location: below elbow (DAVF, *n* = 174) and at the level of elbow or higher (PAVF, *n* = 69). In DAVF group, the percentage of persistent AVF was lower (52% vs. 66%, *p* = 0.2291 for trend) but it did not achieve statistical significance. The baseline characteristics of both groups were presented in Table 2. The graft function and demographic data did not show significant differences between both subgroups. PAVF group has presented a significantly longer dialysis vintage, shorter time from KTx to the visit and higher LVD-36 scores. The proportion of patients with HF and dyspnea was also higher in PAVF group.

**Table 1.** Baseline population characteristics, comorbidities, and symptoms of control and HD-groups.

	Control group (*n* = 41)	Hemodialysis (HD-group) (*n* = 243)	
Variable	Median (IQR) or mean ± SD	Median (IQR) or mean ± SD	*p* Value
Age [years]	47 (41–65)	58 (45–64)	0.1171
Sex [males/females]	22/19	144/99	0.4994
BMI [kg/m^2^]	24.2 (21.5–28.3)	26.3 (23.9–29.1)	0.0241
Serum creatinine [mg/dl]	1.25 (1.06–1.65)	1.45 (1.2–1.66)	0.3324
Dialysis vintage [months]	18 (10–38)	24 (13–38)	0.1369
Time from KTx to visit [months]	92 (61–154)	79 (32–144)	0.0987
LVD-36 score* [%]	6.9 (0–19.4)	25 (11.1–41.7)	0.0001
Comorbidities and prevalence of symptoms [n, %]			
Heart failure	4 (9.7%)	39 (16.1%)	0.4212
Coronary artery disease	2 (4.9%)	39 (16.1%)	0.1005
Diabetes	13 (31.7%)	56 (23%)	0.2410
Smoker	2 (4.9%)	17 (7%)	0.8696
History of smoking	8 (19.5%)	87 (35.8%)	0.0478
Dyspnea	2 (4.9%)	40 (16.5%)	0.0901
Lower extremities edema	8 (19.5%)	72 (29.6%)	0.1247
Fatigue	9 (21.9%)	93 (38.3%)	0.0526
Worse exercise tolerance	16 (39%)	109 (44.8%)	0.5026
Palpitations	7 (17.1%)	61 (25.1%)	0.3253
Number of symptomatic patients	22 (53.7%)	154 (63.4%)	0.2966

*LVD-36 scores were given only for symptomatic patients.

**Table 2.** Baseline population characteristics, comorbidities, and symptoms of HD-groups.

	DAVF (*n* = 174)	PAVF (*n* = 69)	
Variable	Median (IQR) or mean ± SD	Median (IQR) or mean ± SD	*p* Value
Age [years]	53.72 ± 13.16	55.43 ± 11.73	0.5577
BMI [kg/m^2^]	26.07 (23.85–29.4)	26.77 (24.24–29.07)	0.8175
Actual serum creatinine [mg/dl]	1.45 (1.2–1.67)	1.4 (1.14–1.6)	0.3893
Dialysis vintage [months]	22 (13–34)	34 (17–54)	0.0046
Active AVF [n, %]	90 (52%)	46 (68%)	0.2549
Time from KTx to visit [months]	90 (32–156)	65 (32–112)	0.0449
LVD-36 score* [%]	18.1 (8.3–36.1)	40.3 (25–52.8)	<0.0001
Comorbidities and prevalence of symptoms [n, %]			
Heart failure	20 (12%)	16 (24%)	0.0482
Coronary artery disease	26 (14.9%)	13 (19%)	0.5132
Diabetes	34 (20%)	22 (32%)	0.1044
Smoker	7 (4%)	10 (15%)	0.0079
History of smoking	59 (34%)	28 (41%)	0.4856
Dyspnea	20 (12%)	20 (29%)	0.0059
Lower extremities edema	51 (29%)	21 (31%)	0.8753
Fatigue	67 (39%)	26 (38%)	0.9624
Worse exercise tolerance	82 (47%)	27 (40%)	0.5021
Palpitations	42 (24%)	19 (28%)	0.6515
Number of symptomatic patients	108 (62%)	42 (61%)	0.8843

*LVD-36 scores were given only for symptomatic patients.

**Figure 2.**
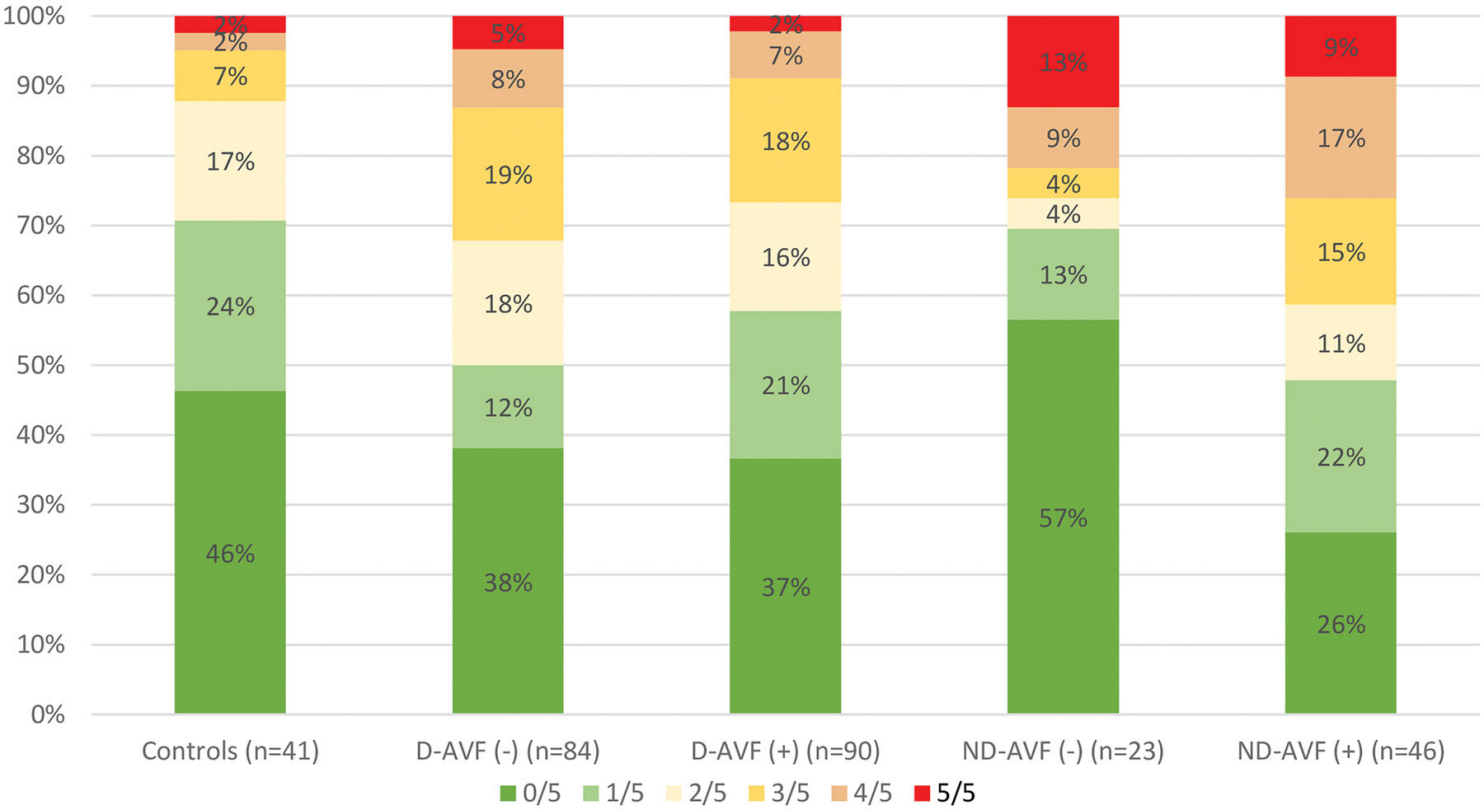
Proportion of patients presenting a certain amount of self-reported symptoms.

To assess the impact of AVF patency and location parallelly, the whole study group was divided then into 4 subgroups according to AVF location and patency: DAVF(−), DAVF(+), PAVF(−), and PAVF(+). Firstly, the number of presented symptoms was analyzed. The proportion of patients who revealed a certain number of symptoms (maximum 5 symptoms) is presented in Figure 2. No statistically significant differences across study groups were noted. In a multivariable logistic regression model, HF, CAD, obesity (BMI >30kg/m^2^) and age remained independent predictors of cardiovascular symptoms’ occurrence in the whole study group (Table 3).

**Figure 3.**
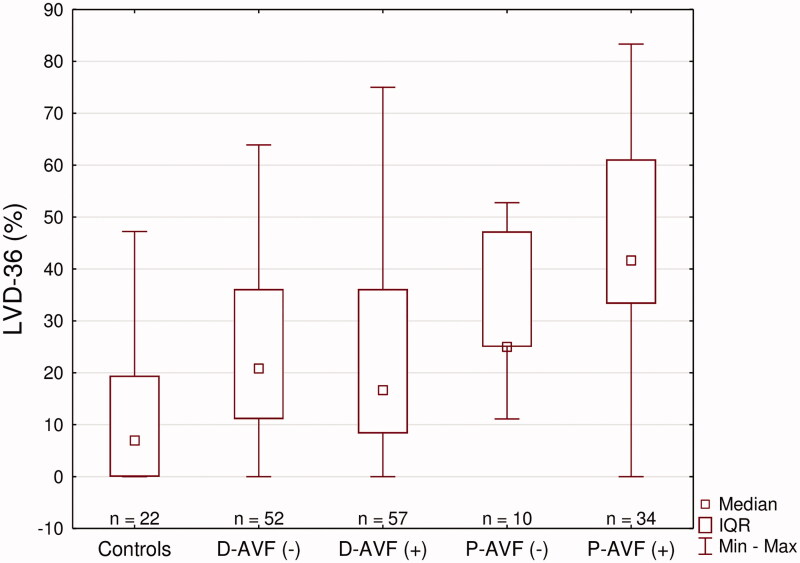
LVD-36 scores among symptomatic patients.

To uncover more discrete differences, LVD-36 scores were analyzed subsequently. Figure 3 presents the ranges of reported scores across these subgroups: the highest value presented by PAVF(+) group, followed by PAVF(−) and both DAVF groups which had the lowest severity of symptoms in HD-group. The control group presented the lowest LVD-36 scores in the whole study. An unadjusted comparison between all presented subgroups is shown in Table 4. All 5 subgroups did not show any other significant differences.

**Table 4.** Unadjusted LVD-36 cross-comparison between all study subgroups (ns for *p* > 0.05, *p ≤ 0.05, **p ≤ 0.01, ***p ≤ 0.001).

Number of patients in each group	LVD-36 score: mean ± SE	Groups	PAVF(+)	PAVF(−)	DAVF(+)	DAVF(−)	Controls
22	12.5 ± 3.12	Controls	***	*	ns	ns	–
52	23.08 ± 2.22	D-AVF(−)	***	ns	ns	–	ns
56	23.11 ± 2.76	D-AVF(+)	***	ns	–	ns	ns
10	31.66 ± 4.76	P-AVF(−)	ns	–	ns	ns	*
34	44.7 ± 3.21	P-AVF(+)	–	ns	***	***	***

*p* Values were multiplicity-corrected.

In a multivariable regression model performed among all symptomatic KTRs, several factors remained independent predictors of the severity of symptoms, including PAVF, serum creatinine levels, and the presence of HF and CAD (Table 5). These findings have been confirmed in a similar multivariate model performed among symptomatic KTRs with DAVF (Table 6).

**Table 5.** Multivariable linear regression model with reverse stepwise elimination for predictors of LVD-36 scores among all symptomatic KTRs with past history of hemodialysis (*n* = 176).

All symptomatic KTRs	Univariable analysis (*p* value)	Multivariable analysis (*p* value)	Coefficient	95% CI
Age [years]	0.0034	–	–	–
BMI [kg/m^2^]	0.0138	–	–	–
Serum creatinine [mg/dl]	<0.0001	0.0435	6.1 / 1mg/dl	0.18 − 12
Active smoker (Yes)	0.0031	–	–	–
AVF status (Active AVF)	0.0018	–	–	–
AVF location (PAVF)	<0.0001	0.0007	7.97	4.89 − 11.06
Heart failure (present HF)	<0.0001	0.0127	4.47	0.97 − 7.97
Coronary artery disease (present CAD)	<0.0001	0.0008	5.85	2.49 − 9.22

R^2^ = 0.3625, 95% CI: 0.252–0.472.

**Table 6.** Multivariable linear regression model with reverse stepwise elimination for predictors of LVD-36 score among previously hemodialyzed symptomatic KTRs with distal AVF (*n* = 110).

DAVF (−) vs. DAVF (+)	Univariable analysis (*p* value)	Multivariable analysis (*p* value)	Coefficient	95% CI
Age [years]	0.0560	0.5048	–	–
Serum creatinine [mg/dl]	0.0333	0.0485	6.42 / 1mg/ml	0.04 − 12.9
AVF status (Active AVF)	0.9984	0.5739	–	–
Heart failure (present HF)	<0.0001	0.0451	4.61	0.1–9.1
Coronary artery disease (present CAD)	<0.0001	0.0005	7.41	3.3–11.5

R^2^ = 0.2514, 95% CI: 0.116–0.386.

**Figure 4.**
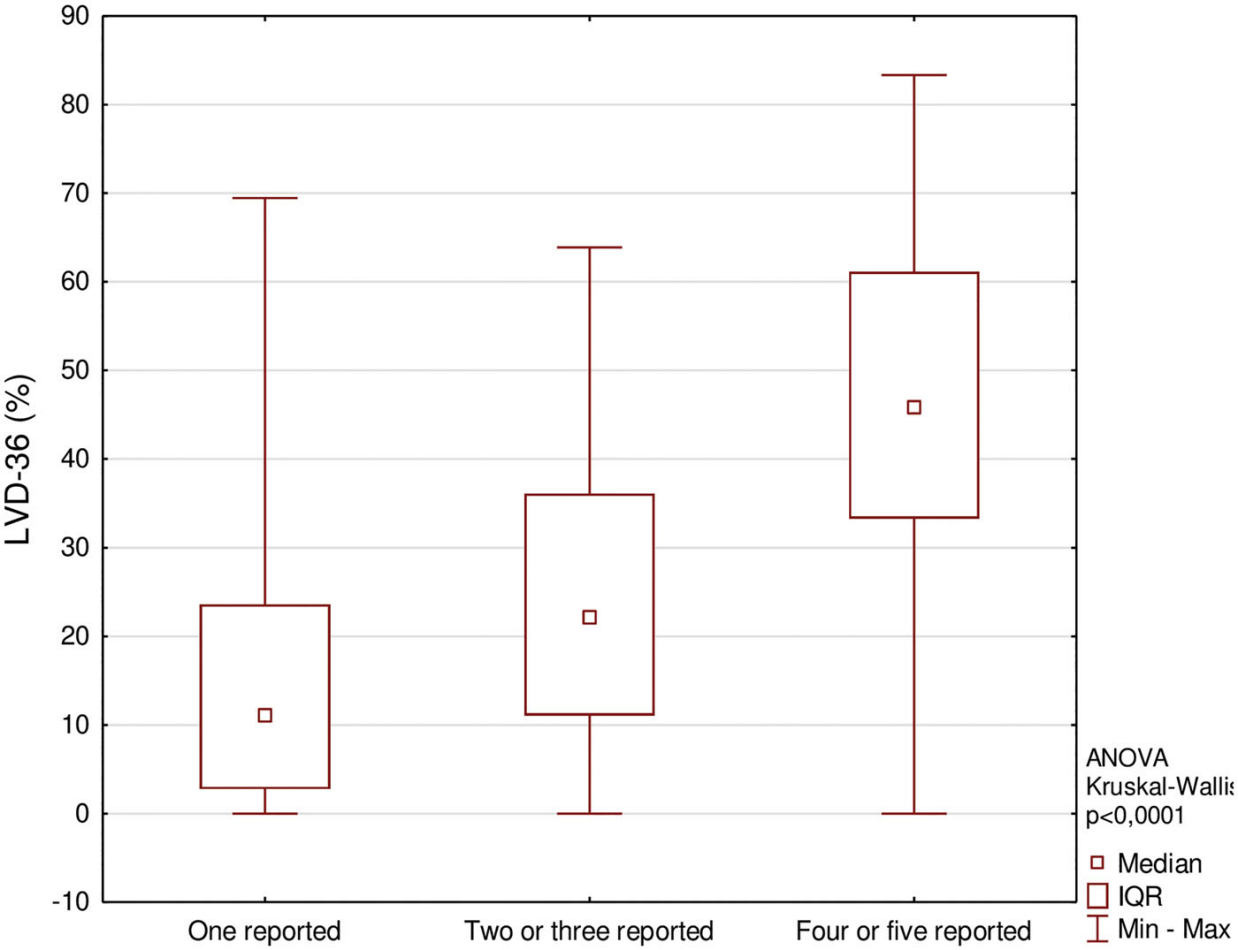
LVD-36 scores according to amount of self-reported symptoms.

**Figure 5.**
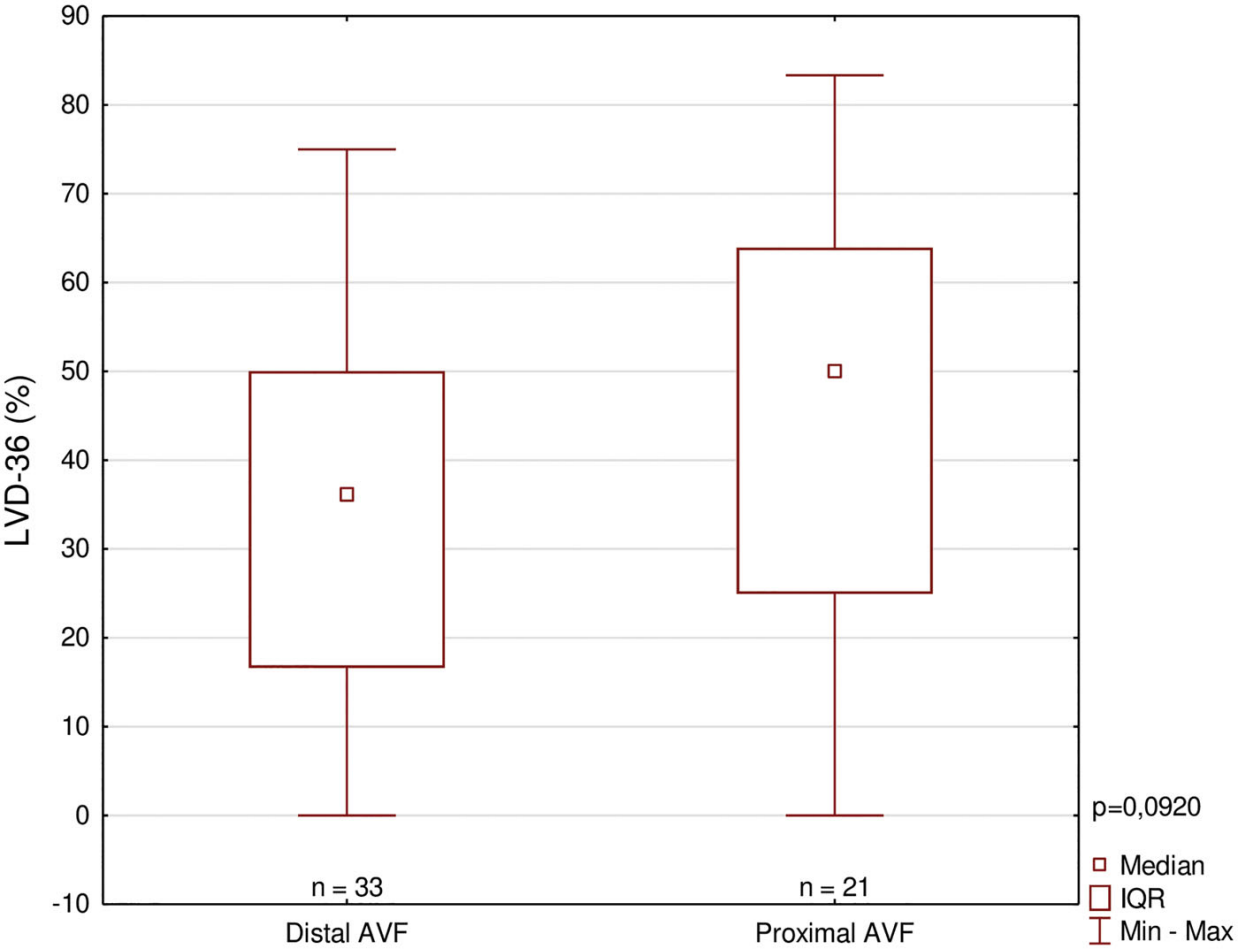
LVD scores among patients with heart disease according to AVF location.

**Figure 6.**
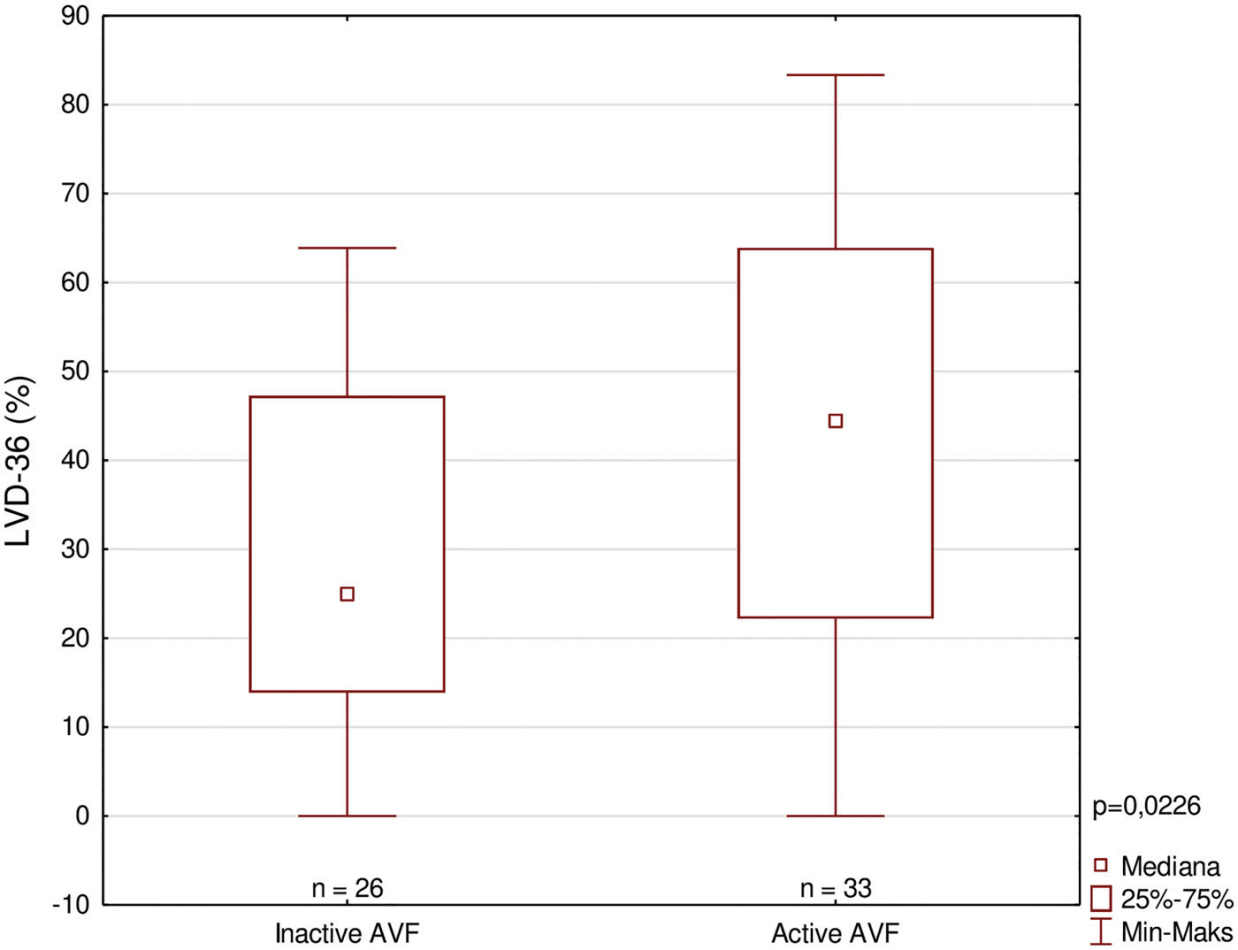
LVD scores among patients with heart disease according to AVF patency.

Figure 4 shows median LVD-36 scores among patients who have reported certain number of symptoms, which indicates an appropriate LVD-36 questionnaire use. We have also aimed to elucidate the impact of AVF location and patency on patients with significant heart disease (defined as either diagnosed CAD or HF, or both of them). These patients were divided according to these parameters, and LVD-36 scores were compared among them (Figures 5 and 6). In patients with significant heart disease, the presence of active AVF was associated with worse function outcome (higher LVD-36 scores). No such difference was noted when analyzing the impact of AVF location.

## Discussion

Nowadays, clinical tools based on patient-reported outcomes (PRO) have found their place in management of patients in several fields of medicine, such as urology, rheumatology, or gastroenterology [16–18]. The clinically reported outcomes, such as, for instance, endpoints (mortality, admissions to hospitals, or complication rates), echocardiographic or biochemical parameters, have been measured strictly by most of the studies assessing the impact and benefits of potential elective AVF ligation. However, none of them has focused on PRO, the significance of which is increasingly underlined in scientific literature [19,20].

As previously mentioned, the main determinants of quality of life in KTRs, which are described in the literature, include age, comorbidities (such as CAD, HF, or diabetes), cold-ischemia time, and dialysis vintage; all of which are directly associated with cardiopulmonary capacity [21]. It has also been shown that the physical activity of KTRs in the early post-transplant period is still lower than in sedentary healthy controls [22]. Nonetheless, factors limiting the activity of KTRs, such as mental disorders or musculoskeletal limitations, may significantly reduce QOL as well.

The cohort of KTRs suffers from many cardiovascular complications. Echocardiographic abnormalities, as well as concentric hypertrophy and concentric remodeling [23], are common findings, resulting in left ventricular dysfunction [24]. Although no major differences in the prevalence of self-reported complaints have been found between the analyzed patient groups in our study, the use of LVD-36 questionnaire allowed to uncover more discrete discrepancies regarding EC in this patient cohort. The results of LVD-36 in HD-group are comparable to scores obtained in patients with HF (either diastolic and systolic) classified as NYHA IIa [25].

The most important factors associated with the presence of cardiovascular symptoms in our study were CAD, HF, and obesity. The presented material also indicates that both the creation of proximal vascular access and persistent patent AVF are associated with worse long-term EC after kidney transplantation, especially in patients with heart disease. In this patient subset, patent AVF remained an independent predictor of worse clinical outcome. That is why patients with PAVF and cardiovascular burden may be best candidates for post-transplant routine AVF ligation or flow reduction.

The influence of AVF location on performance of KTRs is comparable to the one of the presence of HF or CAD. This may be associated with the fact that larger, high-flow AVF may contribute to irreversible cardiac remodeling, aggravation of coronary ischemia and volume overload already before the transplantation as compared to smaller DAVF [11]. Additionally, most respondents of this study had been living with a kidney transplant for many years, before completing the LVD-36 questionnaire. Therefore, it is conceivable that AVF may have functioned for a good number of years in patients living with a kidney transplant, before becoming non-patent.

The evidence on benefits of AVF ligation in KTRs with past history of hemodialysis is limited and the current recommendations are based on expert opinion (Level Of Evidence: C) [14]. Until now, it has been proven that ligation of AVF was associated with myocardial remodeling reversion, as it results in reduction of NT-pro-BNP serum levels as well as improvement in left ventricular hypertrophy (LVH) and left atrial size [7,26]. Both of these echocardiographic parameters have a prognostic value for survival of KTRs [27]. However, the long-term influence of such intervention on the cardiovascular mortality and heart function remains unknown. An alternative approach for access ligation is flow reduction. The banding procedure can be performed precisely using real-time flow ultrasound monitoring by means of a dowel, dilators, or balloons at various sizes [28–30]. Such procedures were shown to reduce effectively the symptoms of AVF-associated HF [28].

On the other hand, a closure of PAVF might limit future vascular access options, although the dialysis return rates are relatively low and equal about 2–3% yearly [31]. Additionally, the AVF ligation procedure under local anesthesia seems to be a safe intervention, which does not affect transplantation outcome [32]. That is why, a ligation of high-flow AVF after transplantation may contribute to cardiovascular risk reduction and QOL improvement, which makes it a viable future strategy for cardiovascular risk management in KTRs [33,34]. In patients where concerns about graft survival are raised, AVF banding might be more beneficial than AVF ligation, as it does not limit future vascular access possibilities. That is why, we believe that flow reduction and low-flow maintenance may be a procedure that balances, in case of high-flow PAVF, cardiac burden and, in case of moribund KTRs and those who are at potential risk of graft loss, the risk of vascular access loss. This hypothesis should, however, be assessed in prospective studies, including AVF-flow measurement, echocardiography, and clinical follow-up.

It is also worth mentioning that patients with PAVF have shown significantly longer dialysis vintage than the rest of the study group. Despite being an acknowledged predictor of post-transplantation outcome, the dialysis duration and LVD-36 score did not show any correlation in our study. However, the longer dialysis period is likely to be associated with malfunction of previous DAVF, which has led to proximal vascular access creation. That is why, the reduction of transplantation waiting time or distal access preservation might be a way to prevent consequences resulting from vascular access creation in a more proximal site (severe LVH and worse QOL). When preparing for dialysis, physicians should also strive to create the most distal vascular access which is deemed feasible. The anatomic snuffbox fistulae seem to be a safe and reliable alternative for wrist and elbow AVF [35–37].

The precise impact of AVF ligation or banding procedure on QOF, allograft function and cardiovascular risk needs to be scrutinized in prospective trials to identify patient cohort who could potentially benefit from such an intervention.

We acknowledge several limitations of our study. Like all observational reports, our study might be subject to nonrandom selection bias. Secondly, LVD-36 score was not correlated with echocardiographic data, as a patient-filled anonymous questionnaire would not be a reliable source of such information. Additionally, the relationship between AVF location and functional well-being may not necessarily be causal since the observation was not based on any controllable intervention. The distinction between DAVF and PAVF was made on the basis of pure anatomic location rather that depending on the type of anastomosed artery (brachial artery vs. proximal radial artery). However, taking into account literature reports [10], a reasonable assumption can be made that in PAVF, where brachial artery is used in a certain amount of patients, statistically higher flow rates can be expected as compared to DAVF.

Nevertheless, we believe that the presented material is of high quality, and the fact that its outcomes rely on patient-reported ones poses a solid base for future research in this field. Further studies focusing on the impact of AVF patency on KTRs’ QOL and functional outcome are already ongoing (NCT04478968). According to our knowledge, this is also the first study investigating the health status in kidney transplant recipients with a non-complicated AVF compared to their counterparts who have lost the AVF in the course of thrombosis.

The influence of persistent PAVF in KTRs seems to be unfavorable, especially when coexisting with CAD or HF. Cardiovascular disease, graft function, and obesity affect the physical capacity of KTRs as well. To reduce the risk of cardiac burden, more distally located AVFs should be created.

Patients with proximally located AVFs seem to be the best candidates for ligation or flow reduction, as both procedures may improve QOL in KTRs. The decision whether to ligate AVF of perform flow reduction should be individual in every subject.

## Ethical approval

The study was approved by the local ethics committee of the Wroclaw Medical University (approval number: KB-775/2018). The research was conducted ethically in accordance with the World Medical Association Declaration of Helsinki.
